# The last year of life for patients dying from cancer vs. non-cancer causes: a retrospective cross-sectional survey of bereaved relatives

**DOI:** 10.1007/s00520-022-06908-8

**Published:** 2022-02-21

**Authors:** Alina Kasdorf, Gloria Dust, Stefanie Hamacher, Nicolas Schippel, Christian Rietz, Raymond Voltz, Julia Strupp, on behalf of CoRe-Net, Holger Pfaff, Christian Albus, Lena Ansmann, Frank Jessen, Ute Karbach, Ludwig Kuntz, Ingrid Schubert, Frank Schulz- Nieswandt, Stephanie Stock

**Affiliations:** 1 Department of Palliative Medicine, Faculty of Medicine and University Hospital Cologne, University of Cologne, Cologne, Germany; 2 Faculty of Medicine and University Hospital Cologne, Institute of Medical Statistics and Computational Biology, University of Cologne, Cologne, Germany; 3 Department of Educational Science and Mixed-Methods-Research, Faculty of Educational and Social Sciences, University of Education Heidelberg, Heidelberg, Germany; 4 Center for Health Services Research, Faculty of Medicine and University Hospital Cologne, University of Cologne, Cologne, Germany; 5 Center for Integrated Oncology Aachen Bonn Cologne Dusseldorf (CIO ABCD), Faculty of Medicine and University Hospital Cologne, University of Cologne, Cologne, Germany; 6 Clinical Trials Center (ZKS), Faculty of Medicine and University Hospital Cologne, University of Cologne, Cologne, Germany

**Keywords:** Cancer, Non-cancer, Last year of life, VOICES questionnaire, End-of-life care, Proxy perspectives

## Abstract

**Purpose:**

To compare health care experiences of patients with cancer or non-cancer diseases in their last year of life.

**Methods:**

A cross-sectional post-bereavement survey was conducted using an adapted German version of the VOICES questionnaire (VOICES-LYOL-Cologne). Differences in the reported experiences were assessed using a two-sided Pearson’s chi-square test and Mann–Whitney *U* test.

**Results:**

We collected data from 351 bereaved relatives. More than half of non-cancer patients were not informed that their disease could lead to death (*p* < 0.001). When this was communicated, in 46.7% of non-cancer and 64.5% of cancer patients, it was reported by the hospital doctor (*p* = 0.050). In all, 66.9% of non-cancer and 41.6% of cancer patients were not informed about death being imminent (*p* < 0.001). On average, non-cancer patients had significantly fewer transitions and hospital stays in their last year of life (*p* = 0.014; *p* = 0.008, respectively). Non-cancer patients were treated more often by general practitioners, and cancer patients were treated more often by specialists (*p* = 0.002; *p* = 0.002, respectively). A substantially lower proportion of non-cancer patients were treated by at least one member of or in the setting of general or specialized palliative care (*p* < 0.001).

**Conclusions:**

Non-cancer patients experience disadvantages in communication regarding their care and in access to specialized palliative care in their last year of life compared to cancer patients. Regarding the assessment of palliative care needs and the lack of communication of an incurable disease, non-cancer patients are underserved. An early identification of patients requiring palliative care is a major public health concern and should be addressed irrespective of diagnosis.

**Trial registration:**

Prospectively registered by the German Clinical Trials Register (DRKS00011925, data of registration: 13.06.2017).

## Background

Palliative care aims to improve the quality of life of patients facing multiple symptoms related to a life-threatening illness and that of their families, with particular emphasis being placed on the assessment and management of symptoms [1]. For some time, palliative care has mainly focused on the care of patients with cancer [2, 3]. Currently, more attention has been devoted to introducing palliative care early in the trajectory disease in patients with non-malignant diagnoses [4]. Demographically, more than twice as many people die from non-malignant causes than from cancer [5]. Still, non-cancer diseases account for the lowest proportion of use in palliative care [6–8]. In Germany, specific data on the current situation of the two groups are hardly available. In the years between 2007 and 2011, only 8.1% of all non-cancer patients received specialized palliative care [8]. Between 2014 and 2016, 80.5% of all patients receiving specialized outpatient palliative care died from cancer [9]. Overall, 68–97% of non-cancer patients have not received any palliative care services, compared to 50% of cancer patients [9]. In comparison, the percentage of non-cancer patients receiving hospice care in the USA was much higher (29.6% in 2018) [10].

Often, the timing of referrals to palliative care for non-cancer conditions is delayed [7, 11]. Patients with non-malignant disease have a less predictable course due to the frequent variability in the progression of their disease [12] and the lack of awareness of palliative care among caregivers and health professionals [13]. Recent findings in the study by Quinn et al. provide important data about the clinical benefits of palliative care in patients with non-cancer diseases, showing that palliative care is associated with reduced acute care service use, mitigation of symptoms, and increased advance care planning in patients with non-cancer diseases [14].

Cancer and non-cancer patients experience some commonalities in symptom patterns and burden. These symptoms include pain, dyspnea, nausea and vomiting, anorexia, fatigue, anxiety, tension, and depression [15, 16]. Burt et al. [17] compared the experiences in the community in the last 3 months of life of older adults dying from cancer and non-cancer causes, using the *Views of Informal Carers—Evaluation of Services* (VOICES) [18]. These commonalities in the prevalence of problems across cancer- and non-cancer patients highlight the need for palliative care to be provided, irrespective of diagnosis [16].

To the best of our knowledge, the differences in health care provision between cancer and non-cancer patients in the last year of life in Germany have rarely been investigated. To address this research gap, the aim of our study was to evaluate the differences between cancer and non-cancer patients who have died in the region of Cologne, Germany.

## Methods

This study is part of a project entitled the “Last Year of Life Study Cologne (LYOL-C)” [19]. A retrospective cross-sectional survey of bereaved relatives representing decedents in the Cologne area (Germany) was conducted between November 2017 and August 2018. We included all relatives, friends, or volunteers if they were 18 years or older and had cared for a person in his/her last year of life (all will be referred to as “informants” hereafter). We excluded deaths in people under 18 years, as well as deaths by accident. The participants were recruited in cooperation with health and social care practitioners. Two recruitment strategies were used to identify potential participants: (i) questionnaire distribution through cooperating health and social care practitioners through client records by mail or personally and (ii) self-selection through public media (newspaper articles, flyers, and posters) [20].

A structured presentation of the last year of life in patients categorized in two groups supports this analysis, focusing on the most important checkpoints of health care provision in the last year of life [21]. Our analysis focuses on differences in communicating an incurable disease (“transition into the last year of life”); transitions across different health care settings in the last year of life; and general, palliative, and hospice care utilization, as well as place of death (“transition into death”). Ethical approval for the study was granted by the Ethics Committee of the University Hospital of Cologne (#17–188). Relatives received written information about the study and data protection and had to give written informed consent for participation.

### Data collection

A modified German version of the VOICES questionnaire (VOICES-LYOL-Cologne) [18, 19] was sent to sampled informants. VOICES-LYOL-Cologne is a validated survey with 106 items to assess the quality of care in the last year of life irrespective of diagnosis [20] We compared the last 12 months of life, irrespective of age. It is reasonable to consider 12 months before death, because the occurrence and intensity of multiple symptoms in cancer and non-cancer patients occurred equally in both disease groups near the end of life [22].

### Data analysis

Statistical analyses were performed using IBM SPSS© version 24.0 (IBM Corporation, Armonk, NY, USA). Patient characteristics are presented as mean ± standard deviation (SD) or absolute and relative frequencies, respectively. The formation of groups (cancer vs. non-cancer) was based on the items categorizing the diagnosis of the participants. Differences in the reported experiences of cancer and non-cancer decedents in the health care provision in the last year of life were assessed using a two-sided Pearson’s chi-square test and the Mann–Whitney *U* test. Presented *p*-values are two-sided and considered significant if < 5%. All variables were summarized using descriptive statistics, with missing data excluded analyzing continuous and dichotomous variables. Due to the explorative character of this study and the small sample size, no adjustment of the significance level *α* was applied. The results are considered for exploratory purposes. To calculate the correlation coefficient *r* for the Mann–Whitney *U* test, the *z*-value and the sample size (*n*) were used [23]. For the Pearson’s chi-square test, *Cramer’s V* was reported.

## Results

A pool of 351 participants was representative with respect to gender (47.9% male) and age (76.5 ± 13.0 years) compared with full data from Cologne (for more information, see Voltz et al. [20]). Characteristics of the patients and their informants divided in two groups are presented in Table 1.

**Table 1:** Characteristics of deceased patients and informants

	Non-cancer	Cancer
	*% (n)*	*% (n)*
Deceased age at death (years)		
18–29	-	0.5 (1)
30–49	-	2.9 (6)
50–69	7.7 (11)	39.2 (82)
70–89	59.9 (85)	51.7 (108)
90 +	32.4 (46)	5.7 (12)
Deceased sex		
Male	38.7 (55)	54.1 (113)
Female	61.3 (87)	45.9 (96)
Deceased ethnic group		
German	97.9 (139)	96.2 (201)
Other	2.1 (3)	3.8 (8)
Deceased family situation^a^		
Had a partner	29.8 (42)	57.9 (121)
Lived together with partner	24.1 (34)	44.0 (92)
Had children	48.9 (69)	47.4 (99)
Lived together with children	9.9 (14)	8.6 (18)
Lived together with someone else	9.9 (14)	3.3 (7)
Lived alone	44.7 (63)	24.4 (51)
Someone else had power of attorney		
Yes	90.8 (129)	87.1 (182)
No	7.7 (11)	10.5 (22)
Do not know	1.4 (2)	2.4 (5)
Illnesses in the last year of life^a^		
Cardiovascular disease	61.3 (87)	26.3 (55)
Neuropsychiatric disease	63.4 (90)	12.0 (25)
Disease of the respiratory system	43.7 (62)	19.6 (41)
Liver or kidney disease	25.4 (36)	12.9 (27)
Diabetes mellitus	16.2 (23)	10.5 (22)
Multimorbidity	78.0 (110)	49.3 (103)
Informant relation to deceased		
Spouse	23.2 (33)	55.5 (116)
Son/daughter	56.3 (80)	27.8 (58)
Sibling	3.5 (5)	6.2 (13)
Son/daughter-in-law	4.2 (6)	1.4 (3)
Parent	0.7 (1)	1.0 (2)
Other relative	8.5 (12)	1.0 (2)
Friend	2.1 (3)	4.3 (9)
Neighbor	-	0.5 (1)
Volunteer	-	1.0 (2)
Other	1.4 (2)	1.4 (3)
Informant age (years)		
18–29	-	1.0 (2)
30–49	9.2 (13)	15.8 (33)
50–69	66.2 (94)	56.0 (117)
70–89	23.9 (34)	26.8 (56)
90 +	0.7 (1)	0.5 (1)
Informant sex		
Male	21.1 (30)	33.5 (70)
Female	78.9 (112)	66.5 (139)

^a^Multiple responses were possible

There were 142 non-cancer patients and 209 cancer patients. Non-cancer patients were significantly older than cancer patients (non-cancer 84.2 ± 9.64 vs. cancer 71.3 ± 12.4 years; *p* < 0.001). Decedents dying of non-cancer causes were more likely to be female (*p* = 0.007, *Cramer’s V* = 0.151) and over 85 years (*p* < 0.001; *Cramer’s V* = 0.450). Cancer patients were more likely to be male and under 85 years. Among the non-cancer-conditions, the main cause of death was due to neuropsychiatric disease clearly predominating by dementia with 40.8% (Parkinson’s disease with 9.2%, multiple sclerosis with 2.1%, and amyotrophic lateral sclerosis with 2.1%), followed by cardiovascular diseases, and disease of the respiratory system, as shown in Table 1. Most of the non-cancer patients had multiple comorbidities. According to the informants, 57.4% of non-cancer patients and 40.0% of cancer patients had been ill for less than 12 months (*p* = 0.003, *Cramer’s V* = 0.169).

### Transition into the last year of life

In all, 60.2% (*n* = 118) of non-cancer patients had not been told that their disease would lead to death, compared to 21.7% (*n* = 198) of cancer patients (*p* < 0.001, *Cramer’s V* = 0.387). Table 2 shows that one-fifth of non-cancer patients were told this information less than a month before death. The information about death being imminent was given to 33.1% of non-cancer patients and 58.4% of cancer patients (*p* < 0.001, *Cramer’s V* = 0.249).

**Table 2:** How long before death was he/she told that the disease would lead to death?

	Non-cancer (*n* = 45)	Cancer (*n* = 148)
Less than 1 week	11.1 (5)	6.8 (10)
At least 1 week but less than 1 month	11.1 (5)	16.2 (24)
At least 1 month but less than 6 months	35.6 (16)	22.3 (33)
At least 6 months but less than 1 year	11.1 (5)	21.6 (32)
1 year or longer	31.1 (14)	33.1 (49)

Data are presented as % (*n*)

Table 3 shows that hospital doctors are predominantly delivering bad news to both groups of patients. Second most frequent, general practitioners (GPs) are mostly informing non-cancer patients that the disease leads to death, while outpatient specialist physicians are mostly informing cancer patients about their approaching death.

**Table 3:** Who told that the disease would lead to death?

	Non-cancer	Cancer	*p*	*Cramer’s V*
Hospital doctor	46.7 (21)	64.5 (91)	0.050	0.156
Outpatient specialist physician	8.9 (4)	17.7 (25)	n. s	n. s
General practitioner	17.8 (8)	5.0 (7)	0.015	0.202
Close relatives/friends	17.8 (8)	9.9 (14)	n. s	n. s
Someone else	8.9 (4)	2.8 (4)	n. s	n. s

Data are presented as % (*n*). Presented *p*-values are from Pearson´s chi square test

### Transitions within the last year of life

Table 4 shows that more than nine out of 10 non-cancer patients were predominantly treated by a GP or cared for by a nursing home in their last year of life, while cancer patients were more often treated by an outpatient specialist physician.

**Table 4:** Health care provider in the last year of life

	Non-cancer	Cancer	*p*	*Cramer’s V*
General practitioner	95.0 (134)	83.4 (171)	0.002	0.177
Outpatient specialist physician	69.3 (97)	84.0 (173)	0.002	0.174
Specialist palliative home care team	22.9 (32)	50.0 (103)	< 0.001	0.273
Outpatient care service	48.2 (67)	43.5 (90)	n. s	n. s
Hospice	5.2 (7)	27.8 (57)	< 0.001	0.283
Nursing home	43.2 (60)	7.7 (15)	< 0.001	0.419
Outpatient hospice service	5.7 (8)	7.2 (15)	n. s	n. s

Data are presented as % (*n*). Presented *p*-values are from Pearson´s chi-square test

Overall, at least one member/setting of general or specialized palliative care was involved in the last year of life with 39.4% of non-cancer patients and 85.6% of cancer patients (*p* < 0.001; *Cramer’s V* = 0.482), which corresponds to a strong effect.

Regarding the last hospital stay, 20.8% of non-cancer patients and 11.3% of cancer patients predominantly spent their time in an intensive care unit (*p* = 0.033; Cramer’s *V* = 0.129). Furthermore, cancer patients are predominantly cared for by a palliative care team (cancer 43.8% vs. non-cancer 4.2%; *p* < 0.001, Cramer’s *V* = 0.426).

As shown in Table 5, non-cancer patients experienced significantly fewer transitions between care settings and hospital stays in their last year of life than cancer patients. Also, in the last month of life, non-cancer patients had significantly fewer transitions.

**Table 5:** Transitions in the last year of life

	Non-cancer (*n* = 102)	Cancer (*n* = 153)	*p*	*r*
Hospital admissions in the last…				
Month	0.44 ± 0.57	0.62 ± 0.65	0.027	0.14
3 months	1.04 ± 0.95	1.27 ± 1.05	n. s	n. s
6 months	1.37 ± 1.23	1.72 ± 1.44	n. s	n. s
9 months	1.59 ± 1.44	1.98 ± 1.57	0.037	0.13
12 months	1.69 ± 1.58	2.22 ± 1.71	0.007	0.17
Hospital stays in the last…				
Month	0.68 ± 0.75	0.85 ± 0.78	n. s	n. s
3 months	1.10 ± 0.96	1.37 ± 1.13	n. s	n. s
6 months	1.45 ± 1.27	1.76 ± 1.46	n. s	n. s
9 months	1.66 ± 1.52	2.01 ± 1.56	0.048	0.12
12 months	1.73 ± 1.61	2.24 ± 1.73	0.008	0.17
Transitions in the last…				
Month	0.75 ± 0.90	0.99 ± 0.93	0.021	0.14
3 months	1.77 ± 1.54	2.18 ± 1.71	n. s	n. s
6 months	2.49 ± 2.12	3.09 ± 2.63	n. s	n. s
9 months	2.94 ± 2.59	3.65 ± 2.90	0.047	0.12
12 months	3.15 ± 2.80	4.10 ± 3.25	0.014	0.15

Data are presented as mean ± standard deviation. Presented *p*-values are from the Mann–Whitney *U* test. For calculating the correlation coefficient *r*, the z-value and sample size (*n*) were used

### Transition into death

Data showed that 35.9% of non-cancer patients and 46.6% of cancer patients died in acute care hospitals, with non-cancer patients predominantly dying in the general or intensive care unit (non-cancer 28.9% vs. cancer 13.5%; *p* = 0.001, Cramer’s *V* = 0.190). Nearly five times as many cancer patients died in hospice, while most non-cancer deaths occurred in the nursing home (Table 6).

**Table 6:** Place of death

	Non-cancer	Cancer	*p*	*Cramer’s V*
At home	32.4 (46)	24.5 (51)	n. s	n. s
Hospital	35.9 (51)	46.6 (97)	n. s	n. s
Hospice	4.9 (7)	26.0 (54)	< 0.001	0.272
Nursing home	24.6 (35)	2.9 (6)	< 0.001	0.332
Other	2.1 (3)	0.5 (0)	n. s	n. s

Data are presented as % (*n*). Presented *p*-values are from Pearson´s chi square test

## Discussion

### Main findings

This study analyzed the possible differences between patients with cancer and non-cancer conditions in their last year of life according to transitions, communication, generalist or palliative and hospice care utilization, and place of death in Germany.

Although communication about the course of disease is an extremely important element of adequate care, non-cancer patients were informed less often about their incurable disease than cancer patients. Only four out of 10 non-cancer patients were entering the last year of life knowing about the lethality of their disease. Non-cancer patients were also less often informed about imminent death than cancer patients.

Our results show that GPs mainly treated non-cancer patients, while cancer patients were mostly consulting an outpatient specialist physician and being treated by palliative care specialists. Interestingly, most often, GPs were the ones who told non-cancer patients that their disease would lead to death, while cancer patients were informed by an outpatient specialist physician. In the literature, GPs discussed significantly more frequently all end-of-life issues, although these were discussed significantly more frequently with cancer patients than with non-cancer patients [24]. The low rate of information by the GPs about the trajectory of the disease, and end-of-life care, could be due to several factors: GP lack of availability (lack of time, absence or not making home visits) and the ambivalence of patients and GPs to discuss a “bad diagnosis” [25]. In Germany, the need for strengthening the collaboration between primary health care providers and specialist palliative care services has already been communicated by previous research [13]. The role of GPs is a significant one (e.g., initiating palliative care for non-cancer patients). Afshar et al. reported that non-cancer patients are predominately cared for by generalist palliative care and describe an urgent need to enhance interprofessional and interdisciplinary work between different health care professionals [26]. As GPs were identified as the major health care providers for non-cancer patients, the aim of future studies should be to determine the role of GPs as gatekeepers for referral in the of non-cancer patients to specialized palliative care teams.

The qualifications of the health care professionals also play a role in the initiation into palliative care structures. There is a need for improvement in the integration of palliative care into education of education and training to increase the proportion of specialist physicians and GPs with advanced training in palliative care in Germany, which is currently too low [27].

It has already been postulated at an international level that palliative care must be offered on a non-indication-specific basis [3, 16]. Both patient groups can essentially benefit from early integration of palliative care [28]. According to estimates, between 40.7 and 96.1% of deaths would benefit from palliative care, regardless of the indication [29]. One in five cancer patients requires specialized palliative care [30]. By adding non-cancer patients to this group, a 79% increase in specialized palliative care services caseload can be expected [3].

Non-cancer patients were identified by German health care experts as target groups with a particular priority for palliative care [31]. There is still an asymmetric distribution of palliative care in non-cancer patients. Regarding the use of palliative care structures, our data show these are still mainly used by cancer patients (such as primary palliative care or specialized outpatient palliative care). Non-cancer patients were also under-represented in hospice, were less likely to receive outpatient hospice services and less likely to be treated in a hospital palliative ward, but more likely to be cared for in a nursing home or in an intensive care unit. Existing literature confirms that non-cancer patients are using hospice services less often than cancer patients [32]. Additionally, non-cancer patients are more frequently treated in an ICU [33].

Despite the higher probability of having been informed about the lethality of the disease, cancer patients have more transitions in their last year of life than non-cancer patients. A systematic review demonstrated that the use of specialized palliative care is associated with a reduction of hospital admissions for cancer patients [34], while our results assume the opposite. The reason for this could be oncological therapies and potential complications of cancer patients necessitating more transitions, while non-cancer patients and health care professionals have more uncertainty about their diagnosis [35], resulting from the unpredictability of the course of disease. In general, non-cancer patients had a longer time course with a much slower and unpredictable decline, allowing services to be organized more easily in the longer timeline available [35]. Also, a lack of written referral policy guidelines, unpredictable course of non-cancer disease, subsequent difficulties with developing referral criteria, and the lack of non-cancer-specific expertise must be considered [36]. With regard to hospitalization in the last month of life, the results of this study differ from previous evidence. Hospital admissions were more frequent in patients with cancer, while other studies indicated that non-cancer patients are hospitalized more often [37]. A link to the country-specific health care system could be assumed for the results of this study.

Previous research indicates people dying from cancer were less likely to die in a hospital than at home [38]. In most populations, the proportion of home deaths [39] and nursing home deaths [40] was higher in patients with cancer than those without. Our data have not confirmed these results. Non-cancer patients were more likely to die in a care home, while cancer patients were more likely to die in hospice. Cancer deaths were strongly associated with the probability of dying in a hospice in other research [40]. Regarding other places of death, no statistically significant differences were found.

Overall, regarding the assessment of palliative care needs and the partial lack of communication of an incurable disease, non-cancer patients are at a particular disadvantage compared to cancer patients. The unpredictability of disease progression and the estimation of prognosis in non-malignant diseases is used as an explanation for the clear underrepresentation of non-cancer patients in palliative care. Clinicians find it challenging to know when patients should be referred to palliative care [8, 16]. A considerable proportion of non-cancer patients had neurological diseases. This highlights the challenge to integrate palliative care for these patient groups, especially for those with, e.g., dementia, multiple sclerosis, or Parkinson’s disease, who still “fall through the net” and do not receive the holistic care that they need [41, 42]. For neurological diseases in particular, the unpredictable course of the disease, long periods of care, and, in some cases, a lack of information about the roles and services of palliative care among health care providers constitute major obstacles to engage palliative care [43]. It is also particularly important to support the informal carers, some of whom have been caring for the patients for years. Most of non-cancer patients in this sample turned over 80, which is not solely due to dementia patients. CHD patients are also older in our sample. We hypothesize that older patients are more vulnerable to experiencing a complex interplay of multiple problems and symptoms in different domains, concerning not only the physical but also the psychological, social, and functional domains [22]. Due to the changes in physiology related to the aging process and the higher prevalence of multimorbidity in this population, we think that these issues might explain a difference in age here.

Despite these challenges, health professionals should be made aware of the need to refer patients to palliative care at an early stage of their care parallel to the standard treatment, based on the needs of patients [22]. Differences in referral criteria and lack of specific expertise may be possible obstacles to the provision of care for non-cancer patients [36]. The difficulties of identifying palliative care needs have been addressed in the previous literature [44], especially for neurodegenerative conditions [42]. Numerous instruments are available to support the identification of both disease groups. A routine use of instruments for proactive identification of patients in their last year of life is recommended, addressing the need for earlier referral to palliative care [26, 45]. It is important to consider the prognostic quality in assessing the expected survival of non-cancer patients, due to the unpredictable nature of non-malignant diseases [46]. Furthermore, systematic and diagnosis-independent consideration and initiation of palliative care options in clinical practice are required [47].

## Strengths and limitations

To the best of our knowledge, there is no analysis of the health care provision in the last year of life in a German urban area with advanced palliative care consultation service and dedicated hospice care structure to date. This paper provides exploratory insight into the differences in care for patients who have died of cancer and non-cancer. Further research is necessary to examine the effects confirmatively. The retrospective proxy design seems appropriate for this type of research [48]. The data from the survey remain relevant and current at the present time, as there have been no legislative amendments since the 2015 Act to improve Hospice and Palliative Care in Germany [49]. The law is intended to provide better access for all terminal illnesses.

The retrospective classification into the examination group was based on the primary diagnosis. It cannot be excluded that patients with a malignant diagnosis, who have heart failure triggering the severe symptoms were classified into the cancer patient group. It was also not known what reasons caused each transition between the health care settings, so it was not possible to directly compare these transitions.

A further potential limitation might lie in the comparisons of the specific features of the German health care system, since some results are not transferable. In addition, the data generated here came from a large German city. Therefore, no statement can be made about rural regions. Existing evidence has so far been consistent in showing a disparity in palliative care of cancer and non-cancer patients [7, 14]. These results are also limited to people with caregivers/relatives. It also must be considered that an opt-in-research format in general could be seen as a confounder, so we were only able to gain insights into the care experiences of relatives who actively consented.

## Conclusions/implications for practice

For the non-cancer patient group, a clear disadvantage was identified, including the gap in access to palliative care and the lack of communication around prognosis, including the information about the imminent death. The results underline the importance of early integration of palliative care for patient groups with non-malignant diseases, reconsidering the need for disease-modifying treatment strategies. Furthermore, since GPs have been identified as key health care providers for the non-cancer patients and access to palliative care is still limited for this patient population, it will be important for both GPs and outpatient disease-specific specialist physicians to avoid fragmentation of care by communicating relevant patient information. And thus achieving higher quality of end-of-life care for patients and their relatives. A further study is needed to explore why the associated specialists (neurology, nephrology, cardiology, respiratory, etc.) are not sufficiently engaged in conversations with their patients in their last months of life. While the GP may know the patient better, or longer, it is the specialist that may need to answer disease-specific questions and provide prognosis.

## Data Availability

The datasets used and/or analyzed during the current study are available from the corresponding author on reasonable request.
